# Phytochemicals Targeting Ferroptosis: Therapeutic Opportunities and Prospects for Treating Breast Cancer

**DOI:** 10.3390/ph15111360

**Published:** 2022-11-05

**Authors:** Xinyi Zhao, Xueni Wang, Yuzhou Pang

**Affiliations:** 1 Guangxi Zhuang Yao Medicine Center of Engineering and Technology, Guangxi University of Chinese Medicine, Nanning 530200, China; 2 CAS Key Laboratory of Tropical Marine Bio-Resources and Ecology, Guangdong Key Laboratory of Marine Materia Medica, South China Sea Institute of Oceanology, Chinese Academy of Sciences, Guangzhou 510301, China

**Keywords:** breast cancer, ferroptosis, phytochemicals, curcumin, quercetin

## Abstract

Ferroptosis, a recently discovered iron-dependent regulated cell death, has been implicated in the therapeutic responses of various cancers including breast cancer, making it a promising therapeutic target to manage this malignancy. Phytochemicals are conventional sources for medication development. Some phytochemicals have been utilized therapeutically to treat cancers as pharmaceutic agents or dietary supplements. Intriguingly, a considerable number of antitumor drugs derived from phytochemicals have been proven to be targeting ferroptosis, thus producing anticancer effects. In this review, we provide a short overview of the interaction between core ferroptosis modulators and breast cancer, illustrating how ferroptosis affects the destiny of breast cancer cells. We also systematically summarize the regulatory effects of phytochemicals on ferroptosis and emphasize their clinical applications in breast cancer suppression, which may accelerate the development of their therapeutic use in breast cancer.

## 1. Introduction

Breast cancer is one of the leading malignant tumors, ranking sixth among female human cancers [1]. On the basis of the expression of hormone receptors estrogen receptor (ER), progesterone receptor (PR), and human epidermal growth factor receptor 2 (HER2), breast cancer is clinically categorized into three primary subtypes: luminal, HER2-positive, and triple-negative breast cancer (TNBC). The luminal subtype is further classified into luminal A (HER2-negative) and B (HER2-positive) categories [2]. The majority of patients with breast cancer have a subtype that expresses ERs and PRs and hence respond to hormone therapy or aromatase inhibitors. HER-2, a protooncogene that codes for a 185-kDa plasma membrane-bound tyrosine kinase receptor, is not expressed in TNBC, nor are ERs or PRs. A multifactorial approach is often required for the treatment of breast cancer, which may be carried out using either local therapy (such as surgery and radiation), systemic therapy (such as chemotherapy, hormone therapy, and targeted therapy), or both [3]. Although these treatments have reduced breast cancer mortality, treatment failure has made it difficult to treat this malignancy due to the development of apoptosis resistance [4]. Meanwhile, chemotherapeutic drugs elicit many unexpected off-target effects. Phytochemicals are one of the promising sources of chemotherapy, for the development of novel therapies for breast cancer due to their potential efficacy and minimal toxicity [5,6,7]. Numerous phytochemicals reportedly target ferroptosis, a regulated type of cell death, which plays essential roles in many carcinomas, including colorectal cancer [8], lung cancer [9], and liver cancer [10]. Ferroptosis is a recently identified mode of cell death characterized by excessive reactive oxygen species (ROS)-induced lipid peroxidation. Recent evidence suggests that the activation of ferroptosis is an efficient method to inhibit breast cancer [11,12].

In 2012, the term ferroptosis was coined to characterize an iron-dependent cell death produced by the oxidation of polyunsaturated fatty acid-containing phospholipids, with redox-active iron and a lack of lipid peroxide repair capacity [13]. Since the first finding of the function of the system X_c_^−^–glutathione (GSH)–glutathione peroxidase 4 (GPX4) pathway in inhibiting ferroptosis, it has been demonstrated that phospholipid hydroperoxides (PLOOHs), a form of lipid-based ROS, are the ferroptosis executioners [14]. System X_c_^−^ is a cystine/glutamate antiporter that acts to import cystine and maintain the equilibrium of the antioxidant system in cells [15]. It is mainly composed of two subunits: solute carrier family 7 member 11 (SLC7A11) and solute carrier family 3 member 2 (SLC3A2). Ferroptosis is caused by lipid peroxidation, which is normally induced by the inhibition of SLC7A11 and GPX4. Cystine is a substrate required for GSH synthesis. Reduced SLC7A11 expression results in cystine depletion, GSH deficiency, and a consequent increase in lipid peroxidation [16]. GPX4, a crucial lipid peroxidation inhibitor, oxidizes GSH to glutathione disulfide (GSSG). Repressing GPX4 diminishes GSH’s capacity to neutralize lipid peroxidation [17]. Peroxidation of membrane phospholipids containing polyunsaturated fatty acids (PUFAs) is fundamental to the execution of ferroptosis [18]. Acyl-CoA synthetase long-chain family member 4 (ACSL4), a member of the ACSL family, links free long-chain fatty acids to CoA to produce PUFA-coenzyme A (CoA), which is subsequently transesterified into phospholipids (PL) to generate PUFA-PL [19]. Free ferrous iron combines with hydrogen peroxide to form hydroxyl radicals, which initiate lipid peroxidation by removing hydrogen from the bis-allylic position of PUFAs [20]. Lipid peroxidation then occurs when oxygen is added to the PUFAs [21]. In short, three factors play a major role in the control of ferroptosis: the production of ROS [22], the neutral interaction between GSH and ROS mediated by GPX4 [23], and the induction of lipid peroxidation under the action of arachidonate lipoxygenases (ALOXs) [24]. According to these mechanisms, ferroptosis is manifested by the accumulation of iron ions, increased levels of ROS, malondialdehyde (MDA), and GSH. Since ferroptosis is distinct from other modes of cell death, its induction effectively kills cancer cells that are resistant to other kinds of regulated cell death, such as apoptosis and autophagy. As a result, ferroptosis may be a new focus for breast cancer therapeutic development. Recent advances made in discovering the role of ferroptosis in treating breast cancer were thoroughly reviewed [25,26,27].

Combined medication therapy is a potential cancer treatment technique, particularly for those conventional chemotherapeutic agents. For example, cisplatin is often used in conjunction with other chemotherapy medications to treat cancers. The unexpected adverse effects brought on by the combination of chemo-drugs, however, can be intolerable for patients with advanced cancer or poor general health [28]. Therefore, the emphasis on cancer therapy has turned to combining phytochemicals with chemo-drugs [29] and discovering innovative delivery strategies [30]. In-depth studies have recently revealed that phytochemicals have the capacity to stimulate the process of ferroptosis, leading to an inhibited growth of cancers. For instance, the flavonoid apigenin has been extensively investigated for its anticancer properties in a variety of malignancies, including breast cancer [31]. Meanwhile, apigenin’s ability to induce ferroptosis in cases of lung cancer, multiple myeloma, and neuroblastoma has been confirmed [32]. Mbaveng et al. [33] conducted research on sauyauxnium chloride, an indoloquinazoline alkaloid derived from the medicinal herb *Araliopsis soyauxii* Engl, and found that it had IC_50_ values below or around 10 μg/mL with regard to 14/18 (77.8%) cancer cell lines, including two drug-resistant breast cancer cells (MDA-MB-231-pcDNA and MDA-MB-231-BCRP). Further experiments in CCRF-CEM leukemia cells confirmed that sauyauxnium chloride could cause ferroptotic cell death. Similarly, another study found that benzophenone epunctanone isolated from *Garcinia epunctata* could cause cell death in a number of cancer cells, including MDA-MB-231-BCRP [34]. Meanwhile, pre-treatment of cells with two ferroptosis inhibitors (ferrostain-1 and deferoxamine) greatly reduced the cytotoxicity of epunctanone over CCRF-CEM leukemia cells. These indications suggest that phytochemicals have the potential to act as cytotoxic candidates for the treatment of breast cancer by inducing ferroptosis.

**Figure 1:**
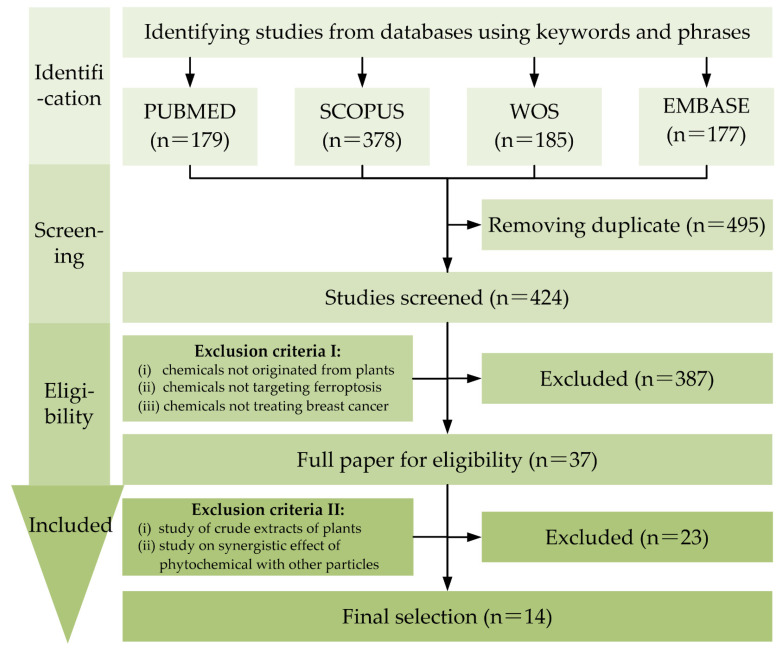
Flowchart of study selection for this review.

In this review, a literature survey was conducted for the period from 1 January 2012 to 30 September 2022. Four databases (Web of Science, Scopus, PubMed, and Embase) were screened. Due to the reason that many articles on phytochemicals addressed only the scientific name of the chemicals, the keywords and phrases used in the search were “breast cancer” AND “ferroptosis”, to ensure that no research on this topic was missed. The initial search yielded 919 articles in the databases (PubMed: 179, Scopus: 378, Web of Science: 185, and EMBASE: 177). After removing the duplicates, 424 publications were screened based on their titles and abstracts. Two sets of exclusion criteria were established for the eligibility after removing duplicates. The first set of exclusion criteria were: (i) chemicals that do not originate from plants, (ii) chemicals that do not target ferroptosis, and (iii) chemicals that are not relevant to breast cancer treatment. The second set of exclusion criteria included: (i) studies of crude extracts of plants, and (ii) studies on the synergistic effect of phytochemicals with other particles. The number of relevant articles that were finalized after extraction and analysis using the criteria above was 14. A workflow diagram depicts our approaches (Figure 1). Here, we summarized the phytochemicals suppressing breast cancer by regulating the onset of ferroptosis. In this way, some patterns are summarized, and this will hopefully be useful for the development of phytochemicals in anti-breast cancer drugs.

## 2. Phytochemicals Suppressing Breast Cancer by Targeting Ferroptosis

### 2.1. 18-β-Glycyrrhetinic Acid

The active compound 18-*β*-Glycyrrhetinic acid (GA) is mainly extracted from the medicinal plant licorice. GA demonstrates excellent cytotoxic, antimicrobial, anti-inflammatory [35], and anticancer [36] effects.

According to Wen et al. [37], GA treatment specifically reduced cell viability and caused ferroptosis in the MDA-MB-231 cells, along with an increase in lipid peroxidation and ferrous ion. GA’s effects were eliminated by the iron chelator deferoxamine mesylate (DFO) and ferroptosis inhibitor Ferrostatin-1 (Fer-1). In addition, GA treatment boosted the generation of ROS/reactive nitrogen species (RNS), which is a mediator of ferroptosis, as well as the expression and activity of NADPH oxidase and inducible nitric oxide synthase (iNOS) in the MDA-MB-231 cells [38]. SLC7A11 of System X_c_^-^ expression, GSH level, and GPX activity were down-regulated by GA. Together, NADPH oxidases, iNOS, and decreased GSH and GPX activity could help GA increase the generation of ROS and RNS, which would then exacerbate lipid peroxidation and cause ferroptosis in TNBC cells.

### 2.2. Alloimperatorin

Alloimperatorin is a representative natural coumarin isolated from *Angelica dahurica*. It is reported that alloimperatorin could alleviate oxidative stress, inflammation, and apoptosis in Piroxicam-induced gastric ulceration and hepatorenal toxicity in an in vivo experiment [39].

Zhang et al. [40] found that alloimperatorin inhibited breast cancer cell viability in a concentration- and time-dependent manner and that ferroptosis inhibitors drastically reduced the alloimperatorin’s cytotoxic effect. Alloimperatorin caused significant mitochondrial shrinkage and increased Fe^2+^, ROS, and MDA accumulation. These changes indicated the activation of ferroptosis, possibly by a significant reduction in mRNA and protein expression levels of SLC7A11 and GPX4. GPX4 overexpression vectors significantly abolished the anti-invasive effect of alloimperatorin on breast cancer cells. This result presents that alloimperatorin inhibits cell proliferation and invasion by inducing ferroptosis in breast cancer.

### 2.3. Curcumin

Curcumin is the main bioactive compound extracted from the *Curcuma longa* underground rhizome which has a variety of biological activities, including tumor prevention and treatment [41]. Emerging studies showed that curcumin could inhibit the viability of glioblastoma [42] and the viability and proliferation of non-small-cell lung cancer [43] via regulating ferroptosis.

Similarly, Li et al. [44] revealed that curcumin could inhibit the growth of breast cancer cells by inducing ferroptotic death. Curcumin-induced cell death is alleviated by the iron chelator deferoxamine and the inhibitor Fer-1. Meanwhile, marked accumulation of intracellular iron, ROS, lipid peroxides, and MDA, and a downregulated level of GSH are found with the application of curcumin. According to a transcriptomic analysis, curcumin upregulates a variety of ferroptosis target genes, including heme oxygenase-1 (HO-1). Curcumin’s induction of ferroptosis in breast cancer cells is stopped by the specific inhibitor zinc protoporphyrin 9 (ZnPP) of HO-1, as shown by improved cell viability, a decrease in intracellular iron ions, and other ferroptosis-related manifestations. These findings show that curcumin-induced ferroptosis in breast cancer cells may be facilitated by HO-1.

According to Cao et al. [45], curcumin has the ability to reduce the survival of breast cancer cell lines MDA-MB-453 and MCF-7, while also hampering the development of xenograft tumors in mice. The application of curcumin resulted in decreased cell viability along with an increase in solute carrier family 1 member 5 (SLC1A5). The inhibition of SLC1A5, an essential transporter of glutamine, reversed the induction of lipid ROS, MDA production, and intracellular Fe^2+^ levels caused by curcumin treatment [45]. SLC1A5 is a subunit of importers SLC1A5/SLC38A1, which transfer glutamine into cell cytoplasm before glutaminase 2 (GLS2) converts it to glutamate (Glu) in the mitochondria. The subsequent conversion of glutamate to a-ketoglutarate (a-KG) by the enzyme glutamic-oxaloacetic transaminase 1 (GOT1) leads to ROS accumulation through an unidentified mechanism, initiating ferroptosis [46].

### 2.4. Dihydroisotanshinone I

Dihydroisotanshinone I (DT) is a phytochemical purified from the widely used medicinal plant Danshen (*salvia miltiorrhiza* Bunge). DT is a member of the tanshinone family, and it shares structural similarities with tanshinone I which is known to promote apoptosis in breast cancer cells [47,48]. It is reported that DT induces ferroptosis in lung cancer cells [49].

The growth of MCF-7 cells and MDA-MB-231 cells is inhibited by DT in a time- and dose-dependent manner, and at the same concentration DT suppresses these cells’ growth more effectively than other therapeutic treatments (oxaliplatin, gemcitabine, and 5-fluorouracil) [50]. DT significantly increases MDA levels and decreases GPX4 activity in breast cancer cells, causing ferroptosis via lipid peroxidation. In vivo experiments with mice xenografted with MCF-7 cells show that intraperitoneal injection of DT for 2 weeks significantly reduces tumor volume by approximately 70% while the mice’s activity and body weight remain unchanged [50]. These findings suggest that DT inhibits breast cancer growth while causing slight side effects.

### 2.5. Eupaformosanin

Eupaformosanin (Eup) is extracted from *Eupatorium cannabinum* Linn. Evidence showed that Eup suppressed TNBC via ferroptosis. In a concentration- and time-dependent way, Eup suppresses the growth of TNBC cells (MDA-MB-231 and MDA-MB-468), leading them to enter the G2/M cell cycle. Lipid ROS accumulation and GSH depletion were observed after the treatment with Eup. Eup also downregulated iron levels in cells, increased intracellular chelate iron, and reduced the negative regulators of ferroptosis GPX4 and ferritin heavy chain 1 (FTH1), while the cell death could be rescued by the administration of exogenous GSH. Additionally, the ferroptosis inhibitors Fer-1, DFO, and liproxstatin-1 (Lip-1) mitigated the cell death produced by Eup. Therefore, the Eup-induced growth suppression in TNBC may be connected to ferroptosis. The Eup significantly stimulates mutant p53 ubiquitination in MDA-MB-231 cells. Due to the high incidence of TP53 mutations, mutant p53 is considered to be a potential diagnostic and therapeutic target in TNBC [51], and it also regulates ferroptosis via transcriptional or post-translational mechanisms [52]. When mutant p53 was suppressed in TNBC cells, Eup-induced ferroptosis decreased, demonstrating that mutant p53 is a crucial target for Eup therapy. A xenograft model in nude mice also indicated that Eup could decrease the expression of mutant p53 and promote ferroptosis, therefore inhibiting the growth of MDA-MB-231-driven tumors [53].

### 2.6. Formosanin C

Formosanin C (FC), a diosgenin saponin extracted from *Cestrum laevigatum* L. [54] or *Cestrum laevigatum* L. [55], was reported to be a ferroptosis inducer in human hepatocellular carcinoma cells [56]. It was then shown that FC enhanced GPX4 depletion, cytosolic and lipid ROS production, and ferroptosis activation in TNBC MDA-MB-231 cells. The application of Fer-1, a ferroptosis inhibitor that quenches ROS, might counteract the inhibitory effects of FC. Furthermore, FC also enhanced the cell growth inhibition of cisplatin in TNBC MDA-MB-231 cells, which highlighted its role in promoting chemosensitivity [57].

### 2.7. Gallic Acid

Gallic acid is a naturally occurring polyhydroxy phenolic molecule which is frequently present in many foods. It has multiple bioactive activities including cardio-protective [58], anti-inflammatory [59], and anti-tumor activities [60]. Khorsandi et al. [61] conducted research on gallic acid in several cancerous cells including MDA-MB-231. MDA-MB-231 represents an increased level of ROS production and a decreased level of GPX4, which can promote the onset of ferroptosis and inflammation. Moreover, MDA production was evaluated in MDA-MB-231. MDA is an end-product of lipid peroxides, which can be a replacement for lipid peroxides as a biomarker in ferroptosis [62]. The loss in GPX4 and the increase in MDA show that gallic acid may be related to the induction of ferroptosis in MDA-MB-231 [61]. Intriguingly, a study concerning colorectal cancer found that gallic acid might bind to targets involved in ferroptosis and control the expression of associated proteins, highlighting the modulatory role of gallic acid in triggering ferroptosis [63].

### 2.8. Levistilide A

Levistilide A (LA) is an anti-fibrosis [64] and antioxidant [65] chemical isolated from *Chuanxiong* Rhizoma. Jing et al. [66] revealed that LA might inhibit the growth of malignancies by controlling the critical cellular processes of ferroptosis. LA dose-dependently decreased breast cancer cell survival and impaired mitochondrial structure and function, possibly through suppressing the expression of GXP4 and downregulating nuclear receptor coactivator 4 (NCOA4). NCOA4 can facilitate ferritinophagy, an autophagic degradation of ferritin that increases the number of ferrous irons [67]. The overexpression of NCOA4 could increase ferritinophagy and accelerate iron-dependent lipid peroxidation and ferroptosis [68]. Other than this mechanism, LA treatment also markedly enhanced ROS-induced ferroptosis via activating the nuclear factor erythroid-2-related factor 2 (Nrf2)/HO-1 signaling pathway since the Nrf2 inhibitor diminished the ferroptosis caused by LA.

### 2.9. Polyphyllin III

Polyphyllin III (PPIII), also known as dioscin, is the most abundant saponin isolated from *Paris polyphylla*. The traditional Chinese medicinal herb *Paris polyphylla* is typically prescribed to treat mastitis, sore throat, and convulsion. It has been demonstrated that polyphyllin I has the ability to inhibit the growth of breast cancer cells [69]. Multiple mechanisms have been hypothesized for the anticancer effects of PPIII, including the activation of cell cycle arrest, apoptosis, and autophagy.

Zhou et al. [70] demonstrated that PPIII exerted its proliferation-inhibitory effect on TNBC cells MDA-MB-231 primarily via ACSL4-mediated lipid peroxidation elevation and activation of ferroptosis. The deletion of ACSL4 mitigates PPIII-induced ferroptosis to some degree. Meanwhile, PPIII treatment triggered a compensatory protective upregulation of System X_c_^−^’s light chain, xCT. xCT, a negative regulator of ferroptosis, is frequently shown to be abnormally overexpressed in cancer cells and is associated with poorer survival results in breast cancer patients [71]. Interestingly, MDA-MB-231 cells became more sensitive to PPIII when being used in conjunction with the xCT inhibitor sulfasalazine. Further evidence comes from the in vivo MDA-MB-231 xenograft model, which shows that the xCT inhibitor significantly increased the suppressive impact of PPIII on MDA-MB-231 cells. This enhancement may be caused by an increase in intracellular lipid peroxidation and ferroptosis. The findings of this work showed that PPIII causes ferroptosis via ACSL4 in MDA-MB-231 breast cancer cells, whereas xCT inhibition made breast cancer cells more sensitive to PPIII.

### 2.10. Quercetin

A flavonoid known as quercetin has been extensively studied in numerous Chinese medicinal herbs, such as lemon peel, onions, and Moringa oleifera [72]. Quercetin is previously reported to have therapeutic potential for various diseases including cardiovascular diseases [73], metabolic diseases [74], and cancers [75].

An et al. [76] found that quercetin could activate ferroptosis in breast cancer cells MCF-7 and MDA-MB-231; this was indicated by increased intracellular levels of iron, MDA, and GSH. Further study showed that quercetin enhanced the nuclear translocation of transcription factor EB (TFEB) protein, upregulating the expression of lysosomal-associated membrane protein 1 (LAMP-1) in breast cancer cell lines, which in turn promoted ferritin degradation and the release of ferric ions, triggering the onset of iron death in these breast cancer cells. The antiproliferation effects of quercetin in breast cancer cell lines can be blocked by TFEB siRNA or chloroquine, an autophagy lysosomal inhibitor. The activation of the lysosomal pathway is involved in ferritinophagy [77]. TFEB regulated lysosomes function in a transcription-dependent way after nuclear transfer by upregulating gene expression related to lysosomal function [78]. An’s study showed that quercetin had the ability to inhibit breast cancer, possibly by mediating TFEB and subsequently activating ferritinophagy.

### 2.11. Robustaflavone 7, 5″-Dimethyl Ether

Robustaflavone 7, 5″-dimethyl ether (RF-A) is extracted from *S. trichoclada* and identified via extensive spectroscopic data. According to Xie et al. [79], RF-A treatment lowered the viability of MCF-7 cells compared to the control group. However, the treatment did not increase the percentage of apoptosis. Further transmission electron microscope (TEM) analysis showed that RF-A-induced cell death occurred through ferroptosis, as indicated by morphological characteristics such as membrane blebbing and chromatin condensation. Meanwhile, the treatment induces a significant accumulation of intracellular ROS in human breast cancer cells, which can be rescued by the presence of ferroptosis inhibitors Fer-1 and DFO. Further study showed that ferroptosis triggered by RF-A is associated with a downregulation of voltage-dependent anion channel 2 (VDAC2). VDAC controls how ions and other molecules move between cellular compartments, whereas VDAC2 is degraded by the E3 ubiquitin ligase Nedd4. It is reported that the Nedd4 expression levels limit the VDAC2/3 protein degradation, increasing the vulnerability of cancer cells to ferroptosis activators [80]. It is demonstrated by the channel’s enhanced expression that ferroptosis is promoted by VDAC2; this results in mitochondrial malfunction and cell death due to lipid peroxidation and ROS generation. Molecular docking showed that the upregulation of VDAC2 was caused by RF-A interaction with Nedd4 [80], which inhibited the elongation of polyUb chains [79]. Molecular docking in another study anticipates the interaction between RF-A and ACSL4, and the results indicate that RF-A might bind to the central hatch domain LEU123 and alter the macromolecular structure of ACSL4 [81].

### 2.12. α-Eleostearic Acid

*α*-eleostearic acid (*α*ESA) belongs to the family of linolenic fatty acids, a naturally rich compound produced by certain plant species including seed oil of the tung tree [82], and is formerly reported to inhibit breast cancer cell growth via apoptosis [83,84].

Beatty et al. [85] found that *α*ESA was a conjugated linolenic acid that could induce ferroptosis in a number of TNBC cell lines, including MDA-MB-468, MDA-MB-231, BT-549, BT-20, and Hs-578T, as a single agent. Their study [85] also suggests that *α*ESA may prevent tumor growth and metastasis in a murine xenograft model. *α*ESA triggered ferroptosis by activating ACSL1, an isoform of ACSLs, instead of the canonical ferroptosis inhibitor GPX4.

## 3. Perspectives

**Figure 2:**
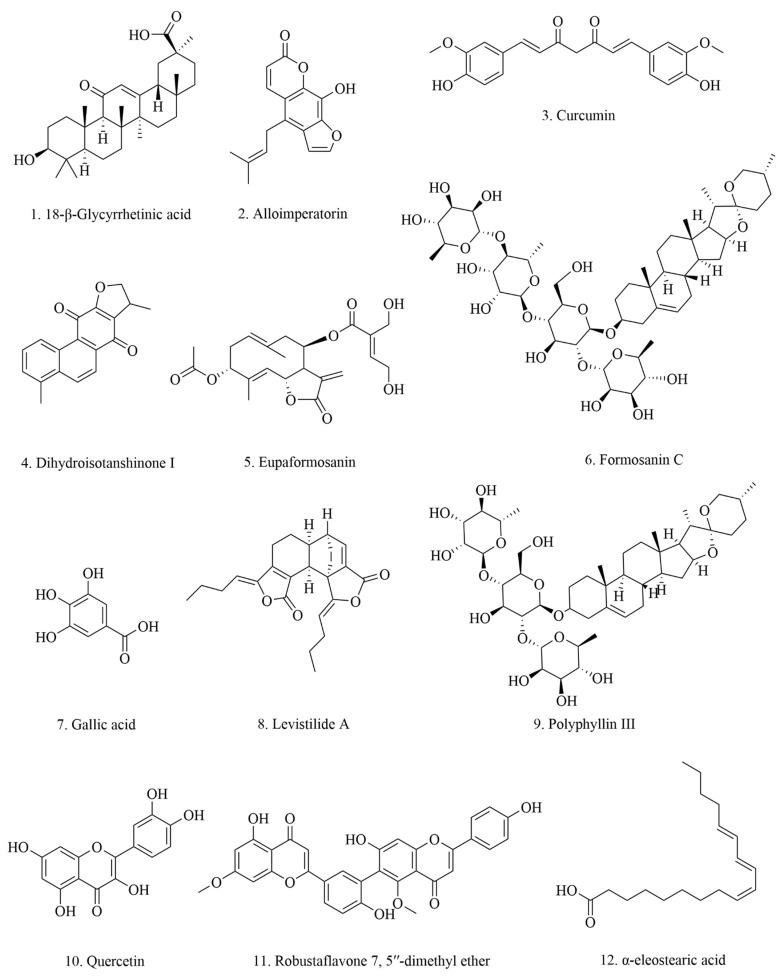
Chemical structures of phytochemicals in Table 1.

**Figure 3:**
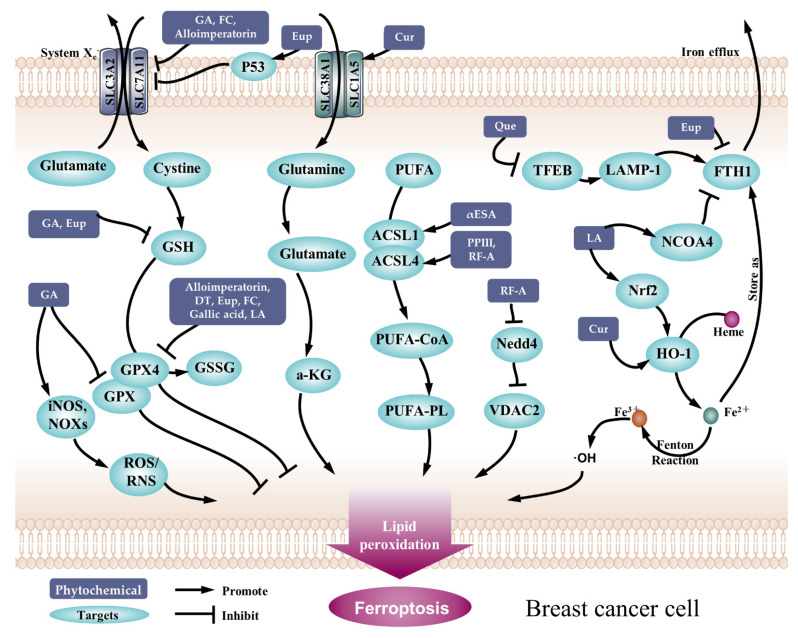
The action mode of phytochemicals inducing ferroptosis in breast cancer cells. **Abbreviations:** ACSL: Acyl-CoA synthetase long-chain family member; a-KG: a-ketoglutarate; CoA: coenzyme A; Cur: curcumin; DT: dihydroisotanshinone I; Eup: eupaformosanin; FC: formosanin C; FTH1: ferritin heavy chain 1; GA: 18-*β*-Glycyrrhetinic acid; GPX: glutathione peroxidase; GSSG: glutathione disulfide; GSH: glutathione; HO-1: heme oxygenase-1; iNOS: inducible nitric oxide synthase; LA: levistilide A; LAMP-1: lysosomal-associated membrane protein 1; NCOA4: nuclear receptor coactivator 4; NOXs: NADPH oxidase; Nrf2: nuclear factor erythroid-2-related factor 2; PUFA: polyunsaturated fatty acids; PL: phospholipids; PPIII: Polyphyllin III; Que: Quercetin; RF-A: robustaflavone 7, 5″-dimethyl ether; TFEB: transcription factor EB; VDAC2: voltage-dependent anion channel 2; αESA: α-eleostearic acid.

Inducing ferroptosis in cancer cells is a potential cancer therapy method. In this review, we collected phytochemicals that could significantly suppress breast cancer cell viability and tumor growth by triggering ferroptosis (Figure 2 and Figure 3). So far, there are only 12 chemicals that meet the criteria for this review. The chemical structure of these compounds is shown in Figure 2. The phytochemicals reported to activate ferroptosis in this review belong to various classifications of chemicals. Among the twelve chemicals, there are four saponins (compounds **4**, **5**, **8**, and **9**), two flavones (compounds **10** and **11**), three organic acids (compounds **6**, **7**, and **12**), one coumarin (compound **1**), one phenylpropanoid (compound **2**), and one quinonoid (compound **3**). The anti-breast cancer effect of compounds **2**, **3**, **4**, **9**, and **12** were verified in animal studies, while the others remain in in vitro studies (Table 1), which require further investigations.

Several phytochemicals discussed in this article have been the subject of clinical trials. Ferry et al. [86] conducted a phase I clinical trial with quercetin on a total of 51 patients with a variety of cancers and discovered quercetin’s antitumor activity. Additionally, it can be administered safely via an intravenous bolus injection. In addition to this study, the clinical trials of these phytochemicals have concentrated on improving the efficacy of chemotherapy or eliminating its side effects. Curcumin, one of the phytochemicals reviewed, was used in combination with chemo-drugs in patients with metastatic and advanced breast cancer. Bayet-Robert et al. [87] undertook an open-label phase I trial to evaluate the feasibility and safety of the combination of docetaxel and curcumin. Fourteen patients with advanced or metastatic breast cancer were assembled. The majority of patients exhibited improved biological and clinical responses. Curcumin should be administered at a dosage of 6000 mg/d for seven consecutive days every three weeks in conjunction with a regular dose of docetaxel. In a randomized, double-blind, placebo-controlled, parallel-group comparative clinical study, Saghatelyan et al. [88] evaluated the effectiveness and safety of intravenous curcumin infusion combined with paclitaxel in 150 patients with metastatic and advanced breast cancer. After 12 weeks of therapy, their study revealed that the combination of curcumin and paclitaxel was superior to the combination of paclitaxel and placebo in terms of objective response rate and physical performance. Curcumin administered intravenously did not result in any serious safety concerns or deterioration of life quality, and it might be effective for alleviating fatigue. In addition, clinical trials demonstrated that curcumin had the ability to alleviate the adverse effects experienced by breast cancer patients following hormonal [89] or radiation therapy [89,90].

Phytochemicals are typically seen as a supplement to traditional chemotherapy as a source of new chemical medicines. However, new data suggested that phytochemicals might play a role in the treatment of drug-resistant cancers. Drug-resistant cells may proliferate during therapy, resulting in therapy failure and subsequently metastasis, which continues to be the primary factor in cancer-related death. Among the 12 phytochemicals in this review, quercetin is thoroughly studied for its ability to treat chemotherapy-resistant breast cancer. El-Kersh et al. [91] reported that quercetin exhibited cytotoxicity in two human estrogen-dependent breast cancer cell lines (MCF-7 and T47D). Additionally, quercetin’s inhibitory activity against the aromatase enzyme was demonstrated in vitro, and lower tumor sizes in mice further support its anticancer potential. There was a decrease in solid tumor aromatase activity in the treatment groups, suggesting an antitumor potential for quercetin. Quercetin was examined in tamoxifen (TAM)-resistant MCF-7 cells and its ability to inhibit Pin1 suggested that it could be useful to treat TAM-resistant breast cancer [92]. A first-line therapy for metastatic breast cancer is the anti-mitotic drug docetaxel. Prieto-Vila et al. [93] discovered that drug resistance was induced in parental cells by upregulating lymphoid enhancer-binding factor-1 (Lef1), a principal regulator of a group of genes whose expression is elevated in drug-resistant cells. Additionally, they confirmed that Lef1 inhibition, especially in treatment with quercetin, re-sensitized cells to docetaxel in a synergistic manner. Therefore, the addition of quercetin to conventional chemotherapy for the treatment of breast cancer may be a potential therapeutic strategy.

Recent years have witnessed a growing number of studies focused on the function of ferroptosis in breast cancer. Ferroptosis has been shown to reduce the growth and progression of breast cancer, as cancer cell death is prevented by Fer-1 or DFO. Conversely, ferroptosis inducers increase ROS, iron, and breast cancer cell death [94]. As a rather newly discovered regulated cell death, ferroptosis and its related genes have been shown in human cohorts to have strong predictive performance in the survival rate and prognosis of patients with breast cancer [95]. The phytochemicals in this review regulate several participants of ferroptosis and exert anticancer effects. The most common target of these phytochemicals is GPX4. Alloimperatorin, DT, Eup, FC, gallic acid, and LA can induce ferroptosis in breast cancer cells by decreasing the level of GPX4. It has been observed that the expression of GPX4 is higher in breast cancer tissues than in normal tissues, and that is adversely correlated with patients’ prognoses [96]. Studies demonstrated with in vitro and in vivo experiments that GPX4 is an oncogene; inhibiting it enhanced the anticancer effects of cisplatin [96]. Furthermore, nanoparticles or drugs targeting GPX4 suppressed TNBC development with few adverse effects [11]. GA, FC, and alloimperatorin downregulate SLC7A11, thus enhancing ferroptosis. Similarly, research on various diseases demonstrate that sorafenib mostly causes ferroptosis by inhibiting SLC7A11 [97,98]. SLC7A11 expression is significantly negatively correlated with the prognosis of patients with breast cancer, indicating that suppressing the negative regulator of ferroptosis may be a viable breast cancer therapy [99]. RF-A, PPIII, and αESA stimulate ferroptosis via upregulating levels of ACSLs. As a critical part of the process of lipid peroxidation, ACSLs are frequently deregulated in cancers [100]. Inhibition of ACSL4 by pharmacological agents ameliorated ferroptotic cell death in a mouse model of TNBC, demonstrating that ACSL4 augmentation is a feasible therapeutic approach for increasing ferroptosis [101]. In addition to targeting these common regulators of ferroptosis, other ferroptotic therapeutic techniques such as CSOSS-Cy7-Hex/SPION/Srfn [102], erastin@FA-exo [103], HMCMs [104], and DFTA [105] have been created and used in breast cancer therapy research. It is plausible to assume that patients with breast cancer may have a better prognosis thanks to phytochemical-induced ferroptotic therapies.

In this review, we comprehensively reviewed the anti-breast cancer action exerted by phytochemicals through triggering ferroptosis. Additionally, this in-depth analysis of different compounds showed that medicinal herbs promise a huge anticancer potential. It is worth noting that the research of these 12 phytochemicals basically fall on the time of 2020 to 2022, indicating the novelty of this field. It is highlighted that these surveyed chemicals play an important anticancer role by targeting diverse ferroptosis regulators (Figure 3), such as GPX4, SLC7A11, HO-1, and FTH1, thus triggering the onset of ferroptosis. In conclusion, the use of bioactive compounds derived from plant sources is an interesting medicinal procedure. When utilized in the treatment of various subtypes of breast cancer, however, extensive information on their effects derived from diverse types of experimental models is necessary.

## Figures and Tables

**Table 1:** Phytochemical compounds modulate ferroptosis in treating breast cancer.

No.	Phytochemical Compound	Molecular Formula	Main targets	Cell Lines	Cell Experimental Concentrations and Durations	Animal Experimental Concentrations	References
1	18-*β*-Glycyrrhetinic acid	C_30_H_46_O_4_	NADPH oxidase, iNOS, SLC7A11, and GPX	MDA-MB-231, MCF-10A, and BT-549	0, 20, 40, 80 μM; 24, 48, and 72 h	N.D.	[37]
2	Alloimperatorin	C_16_H_14_O_4_	SLC7A11 and GPX4	MCF-7 and MDA-MB-231	25, 50, 100, 150, 200 μM; 12, 24, 48, and 72 h	N.D.	[40]
3	Curcumin	C_21_H_20_O_6_	HO-1	MCF-7 and MDA-MB-231	5, 10, 20, 40, 60, 80, 100, 120, and 140 μM; 24 and 48 h	N.D.	[44]
			SLC1A5	MDA-MB-453 and MCF-7	0, 1, 2, 5, 10, 20, and 50 μM; 48 h	30 mg/kg; BALB/c nude mice	[45]
4	Dihydroisotanshinone I	C_18_H_14_O_3_	GPX4	MCF-7 and MDA-MB-231	5 and 10 μM; 24 and 48 h	30 mg/kg; BALB/c-nu female nude mice	[50]
5	Eupaformosanin	C_22_H_28_O_8_	GPX4, FTH1, p53, and SLC7A11	MDA-MB-231 and MDA-MB-468	0, 2, 4, 6, 8, 16 μM; 24, 48, and 72 h	15 mg/kg; BALB/c nu/nu female mice	[53]
6	Formosanin C	C_51_H_82_O_20_	SLC7A11 and GPX4	MDA-MB-231	2, 5, and 10 μM; 24 h	N.D.	[57]
7	Gallic acid	C_7_H_6_O_5_	GPX4	MDA-MB-231	0, 10, 25, 50, 75, 100, and 200 μg/mL; 24 h	N.D.	[61]
8	Levistilide A	C_24_H_28_O_4_	Nrf2, HO-1, GPX4, and NCOA4	MCF-7 and MDA-MB-231	0.625, 1.25, 2.5, 5, 10, 20, 40, 80 μM; 24, 48, and 72 h	N.D.	[66]
9	Polyphyllin III	C_45_H_72_O_16_	ACSL4	MDA-MB-231	2.5, 5, 7.5, 10, 12.5, 15 μM; 24, 48, and 72 h	5 mg/kg; BALB/C nude mice	[70]
10	Quercetin	C_15_H_10_O_7_	TFEB	MCF-7 and MDA-MB-231	0.1, 1, and 10 μM; 24 h	N.D.	[76]
11	Robustaflavone 7, 5′′-dimethyl ether	C_30_H_18_O_10_	VDAC2, Nedd4, and ACSL4	MCF-7	1, 10, 20, 30, 50, 70, 100, and 200 μM; 48 h	N.D.	[79] [81]
12	*α*-eleostearic acid	C_18_H_30_O_2_	ACSL1	MDA-MB-468, MDA-MB-231, BT-549, BT-20, Hs-578T	10, 20, and 40 μM; 24 h	100 µL Tung oil (Sigma-Aldrich 440,337); female NOD. CgPrkdc^scid^Il2rg^tm1Wjl^/SzJ (NSG) mice	[85]

Note: N.D. represents not detected.

## Data Availability

Data sharing not applicable.
